# *Mycobacterium riyadhense* Pulmonary Infection, France and Bahrain

**DOI:** 10.3201/eid1801.110751

**Published:** 2012-01

**Authors:** Sylvain Godreuil, Hélène Marchandin, Anne-Laure Michon, Mikael Ponsada, Georges Chyderiotis, Patrick Brisou, Abdul Bhat, Gilles Panteix

**Affiliations:** Institut National de la Santé et de la Recherche Médicale Unité 1058, Montpellier, France (S. Godreuil);; Hôpital Arnaud de Villeneuve, Montpellier (S. Godreuil, H. Marchandin, A.-L. Michon);; Université Montpellier 1, Montpellier (H. Marchandin, A.-L. Michon);; Laboratoire Biomnis, Lyon, France (M. Ponsada, G. Chyderiotis, G. Panteix);; Hôpital d'Instruction des Armées Sainte-Anne, Toulon, France (P. Brisou);; Awali Hospital, Awali, Bahrain (A. Bhat)

**Keywords:** non-tuberculous mycobacterium, tuberculosis and other mycobacteria, bacteria, pulmonary infection, emerging pathogen, France, Bahrain

**To the Editor:** *Mycobacterium riyadhense* is a newly described mycobacterial species that is potentially pathogenic for humans. Extrapulmonary infection with this nontuberculous mycobacterium (NTM) has been reported (*1*).We report 2 cases of pulmonary infection with this NTM.

The first case of infection was in a 39-year-old woman who was admitted to Toulon Military Hospital, Toulon, France, in December 2005 with suspected pulmonary tuberculosis. For 1 month, the patient had a persistent cough, fever, asthenia, and weight loss. Findings on chest radiographs were suggestive of tuberculosis, with cavitation in the right upper lobe, and the tuberculin skin test reaction was positive. Sputum specimens collected on 3 consecutive days were negative for acid-fast bacilli (AFB), but broth cultures (BacT/ALERT 3D system; bioMérieux, Marcy l’Etoile, France) yielded mycobacterial growth.

We used 4 multiplex line-probe assays to identify the mycobacteria: GenoType MTBC (Hain Lifescience, Nehren, Germany) identified the organisms as members of the *M. tuberculosis* complex (MTBC; with a nonspecific reaction, banding pattern 1, 2, 3); GenoType Mycobacterium CM (Common Mycobacteria) (Hain Lifescience) kit and GenoType Mycobacterium AS (Additional Species) (Hain Lifescience) kit identified the strains as members of the MTBC and as unspecified *Mycobacterium* species, respectively; and INNO-LiPA MYCOBACTERIA v2 (Innogenetics, Ghent, Belgium) yielded a *Mycobacterium*-positive reaction by genus probe but no species-specific result.

Following the criteria of the American Thoracic Society, we considered the isolates as the pathogens responsible for the patient’s respiratory disease (*2*). The patient was treated with a combination of isoniazid (INH), rifampin (RIF), ethambutol (EMB), and pyrazinamide (PZA). EMB and PZA were continued for 2 months; INH and RIF were continued for 10 months (Table), at which time the patient was considered cured.

**Table:** Clinical characteristics, drug susceptibility testing, and outcome for 3 case-patients with *Mycobacterium riyadhense* infection, France and Bahrain*

Patient age, y/sex	Clinical situation	Molecular-based identification of *M. riyadhense*			Drug susceptibility pattern, drug (MIC, µg/mL) interpretation	Antimicrobial drug therapy	Treatment duration, outcome
		Gene	% Sequence similarity with type strain	GenBank accession nos.			
19/M†	Bone infection in left maxillary sinus	16S rRNA, *rpoB*, *hsp65*	Type strain	EU27464, FJ786256, EU921671	AMK (10.0) R; CYC (20.0) S; CIP (2.0) S; CLF (<0.5) S; CLR (<2.0) S; EMB (5.0) S; INH (1.0) I; PAS (>1.0) R; PRO (<1.0) S; RFB (0.2) S; RIF (0.2) S; STR (5.0) S‡	INH, RFP, EMB; then INH, RFP	9 mo, cured
39/F§	Pulmonary infection	16S rRNA¶, *rpoB*, *hsp65*	99.8, 99.8, 100	JF896094, JF896096, JF896098	AMK (<1.0) S; CIP (1.0) S; CLR (0.12) S; DOX (16.0) R; EMB (<0.5) S; ETH (0.3) S; INH (0.5) S; LZD (<1.0) S; MOX (<0.12) S; RFB (<0.25) S; RIF (<0.12) S; STR (1.0) S; TMP/SMX (<0.12/2.38) NA#	INH, RFP, EMB, PZA; then INH, RFP	1 y, cured
43/M**	Pulmonary infection	16S rRNA¶, *rpoB*, *hsp65*	99.8, 99.7, 99.1	JF896095, JF896097, JF896099	AMK (<1.0) S; CIP (0.12) S; CLR (0.12) S; EMB (<0.5) S; ETH (0.3) S; DOX (4.0) R; INH (0.25) S; LZD (<1.0) S; MOX (<0.12) S; RFB (<0.25) S; RIF (<0.12) S; STR (<0.5) S; TMP/SMX (<2.0/38.0) NA#	CLR, CIP; then INH, RFP, EMB, PZA, CLR, CIP; then INH, RFP, CLR, CIP	1 y, relapse; 8 mo, cured

*AMK, amikacin; R, resistant; CYC, cycloserine; S, susceptible; CIP, ciprofloxacin; CLF, clofazimine; CLR, clarithromycin; EMB, ethambutol; INH, isoniazid; I, intermediate; PAS, para-aminosalicylate sodium; PRO, prothionamide; RFB, rifabutin; RIF, rifampin; STR, streptomycin ; RFP, rifapentine; DOX, doxycycline; ETH, ethionamide; LZD, linezolid; MOX, moxifloxacin; TMP/SMX, trimethoprim/sulfamethoxazole; NA, not available; PZA, pyrazinamide. †Patient in Saudi Arabia; reported by van Ingen et al. (*1*). ‡Drug susceptibility testing was performed by using the agar dilution method. §Patient in France. ¶Low 16S rRNA gene polymorphism between several mycobacterial species. #Drug susceptibility testing was performed by using broth microdilution panels (SLOMYCO Sensititer; Trek Diagnosis Systems, Cleveland< OH, USA) and interpreted according to standards of the National Committee for Clinical Laboratory Standards (*3*). **Patient in Bahrain.

The second case of infection was in a 43-year-old man who was admitted to Awali Hospital, Awali, Bahrain, in November 2006. The patient reported malaise, insomnia, cough, weight loss, and anorexia. Radiographs showed features suggestive of tuberculosis (left upper lobe consolidation with focal cavitation). Sputum specimens collected on 3 consecutive days were positive for AFB and mycobacterial growth. To identify the pathogen(s), we used the same 4 mutiplex line-probe assays as used for case-patient 1, and results were similar. The identified strain was considered to be the pathogen responsible for the respiratory disease (*2*).

The patient was treated with a combination of clarithromycin (CLR) and ciprofloxacin (CIP) for 12 months; however, he had a clinical and microbiological (i.e., positive for AFB and culture results with the same NTM) relapse during this treatment. In November 2007, 3 sputum specimens from the patient were positive for AFB, and cultures yielded a mycobacterial strain identical to that identified by the assays. The patient was treated with antituberculous drugs (INH, RIF, EMB, PZA, plus CLR and CIP) for 6 months, and then INH, RIF, CLR, CIP were continued for 2 additional months (Table), after which the patient showed clinical improvement.

In the 2 cases, molecular identification of the isolates as *M. riyadhense* was achieved by using partial *hsp65* and *rpoB* gene sequencing, which was based on the high level of sequence identities with the type strain of *M. riyadhense* and a distance score of 3.5 and 4.6, respectively, to the next species, “*M. simulans*” (Table). Broth microdilution panels (SLOMYCO Sensititer; Trek Diagnosis Systems, Cleveland, OH, USA) were used to determine drug susceptibility (Table) (*3*).

Commercial probes are frequently used for rapid identification of mycobacterial species (*4*); however, *M. riyadhense* and other recently proposed NTMs (e.g., *M. kumamotonense* and “*M. simulans*”) cross-react with MTBC DNA probes and may be missed by line-probe assays (*5**,**6*). With the emergence of new NTM species, commercial probes could fail to discriminate between species, leaving clinical isolates either unidentified or misidentified. Because of its ease of use, accuracy, and discriminatory power, multilocus sequence analysis may soon become the standard for routine NTM species identification.

We have shown evidence for the pathogenic role of *M. riyadhense* in pulmonary diseases, a pathogen that has previously been reported to have extrapulmonary pathogenicity (*1*). Clinical and radiologic signs and symptoms of pulmonary infection caused by *M. riyadhense*, including cough, weight loss, fever, and cavitating lung lesions, were similar to those in typical cases caused by MTBC strains. van Ingen et al. (*7*) suggested that the region of difference 1 (RD1) virulence locus identified in MTBC members may also play a crucial role in virulence of some NTM species. These authors found RD1 genes in NTMs that were causing human disease, including *M. kansasii*, *M. szulgai*, *M. marinum*, and the type strain of *M. riyadhense* (*7*).

We confirmed the presence of RD1 *esat-6* and *cfp-10* genes in the *M. riyadhense* isolates reported here (GenBank accession nos. JF896090–JF896093). Because *M. riyadhense* is an emerging pathogen with, to our knowledge, only 1 previously reported extrapulmonary case of infection (*1*), the optimal treatment for infected patients is unknown. Our results and drug susceptibility testing indicate that antituberculous drugs, including INH, RMP, and EMB, are effective against *M. riyadhense* infection (Table), but the combination of CLR plus CIP was not effective in 1 case-patient reported here, despite in vitro susceptibility to both drugs.
